# Supplementary material for: GRP75 Modulates Endoplasmic Reticulum–Mitochondria Coupling and Accelerates Ca2+-Dependent Endothelial Cell Apoptosis in Diabetic Retinopathy
Source: Biomolecules. 2022 Nov 29;12(12):1778. doi: 10.3390/biom12121778 (PMC9776029; doi:10.3390/biom12121778)
Supplement: Supplementary file 1 [file biomolecules-12-01778-s001.zip › biomolecules-1985720-SI.pdf]

Supplementary materials

Table S1. Main materials and reagents.

Reagents	Source	Identifier
Glucose	Sigma-Aldrich	G8270
Streptozotocin	Sigma-Aldrich	S0130
Evans Blue	Sigma-Aldrich	E2129
AGEs	BioVision	2221-10
Tunicamycin	Abcam	ab120296
BAPTA-AM	MCE	HY-100545
4-Phenylbutyric acid	MCE	HY-A0281
Protein A/G Magnetic Beads	MCE	HY-K0202
Mito-Tracker Green	Beyotime	C1048
Mito-Tracker Red	Beyotime	C1035
ER-Tracker Red	Beyotime	C1041
MPTP assay kit	Beyotime	C2009S
Cell counting kit-8	Beyotime	C0043
JC1 assay kit	Beyotime	C2006
Cell mitochondria isolation kit	Beyotime	C3601
Calcein-AM/PI assay kit	Beyotime	C2015M
Penicillin-Streptomycin solution	Beyotime	C0222
RIPA lysis buffer	Beyotime	P0013B
Rhod-2 AM	YEASEN	40776ES72
TUNEL assay kit	Roche	12156792910
HE staining kit	BOSTER	AR1180
IHC staining kit	BOSTER	SV0004
DAB	BOSTER	AR1027
PAS/Hematoxylin staining kit	Solarbio	G1281
Fetal Bovine Serum	Gibco	10099-141
Trypsin-EDTA (0.25%)	Gibco	25200072
Dulbecco's Modified Eagle Medium	Gibco	C11995500BT
riboFECT CP Transfection Kit	Ribo	C10511-1
GRP75 siRNA Kit	Ribo	stB0006736
Rabbit monoclonal anti-GRP75	CST	#3593
Rabbit monoclonal anti-Bcl-xL	CST	#2764
Rabbit monoclonal anti-Bax	CST	#5023
Rabbit monoclonal anti-Cytochrome c	CST	#11940
Rabbit monoclonal anti-Cleaved Caspase-3	CST	#9661
Mouse monoclonal anti-CHOP	CST	#2895
Normal Rabbit IgG	CST	#2729
Rabbit monoclonal anti-Brn3a	Abcam	ab245230
Rabbit polyclonal anti-4-HNE	Abcam	ab46545
Mouse monoclonal anti-3-NT	Santa	sc-32757
Mouse monoclonal anti-Rhodopsin	Santa	sc-57432
Mouse monoclonal anti-8-OHDG	Santa	sc-393871
Mouse monoclonal anti-VEGF	Santa	sc-7269
Mouse monoclonal anti-IP3R1	Santa	sc-271197
Mouse monoclonal anti-VDAC1	Santa	sc-390996
Normal mouse IgG	Santa	sc-2025
Rabbit polyclonal anti-Bcl-2	Proteintech	12789-1-AP
Rabbit polyclonal anti-COX4	Proteintech	11242-1-AP
Mouse monoclonal anti-β-actin	Proteintech	66009-1-Ig
Goat Anti-Mouse IgG(H+L)	Jackson	115-035-003
Goat Anti-Rabbit IgG (H+L)	Jackson	111-035-003
MitoSOX™ Red	Thermo Fisher	M36008
Pierce™ IP lysis buffer	Thermo Fisher	87787
Goat anti-Mouse IgG (H+L), Alexa Fluor 555	Thermo Fisher	A-21422
Goat anti-Rabbit IgG (H+L), Alexa Fluor 555	Thermo Fisher	A-21428
Goat anti-Mouse IgG (H+L), Alexa Fluor 488	Thermo Fisher	A-11001
DAPI	Thermo Fisher	R37606
ECL kit	Thermo Fisher	34580

Table S2. The sequences of the siRNA.

Product Number	Product Name	Serial Number
stB0006736A	si-GRP75-001	GCGATATGATGATCCTGAA
stB0006736B	si-GRP75-002	GAGTCAGATTGGAGCATTT
stB0006736C	si-GRP75-003	GCTGGAATGGCCTTAGTCA
siB161011044323	NC-siRNA	GGCTCTAGAAAAGCCTATGC

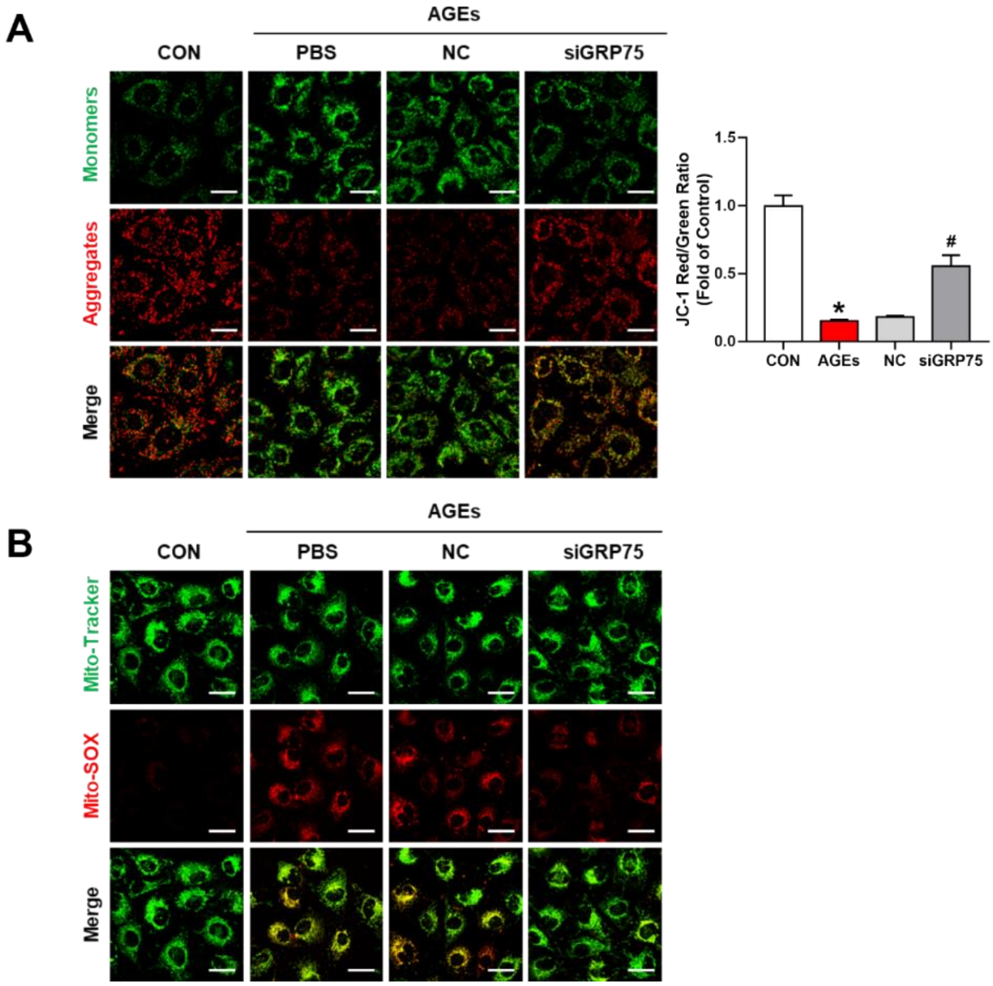

Figure S1. (A) Mitochondrial membrane potential assessment was performed by JC-1 staining. The ratios of red fluorescence (JC-1 aggregates) and green fluorescence (JC-1 monomers) were quantified. Scale bars = 25 μm. **(B)** MitoSOX™ Red (5 μM, 10 min) and Mito-Tracker Green (200 nM, 30 min) was used to detect mitochondrial ROS co-localization with mitochondrial, Scale bars = 25 μm. Results are displayed as means ± SD. *$p < 0.05$ *vs*. control group, $^{\#}p < 0.05$ *vs*. AGEs group.

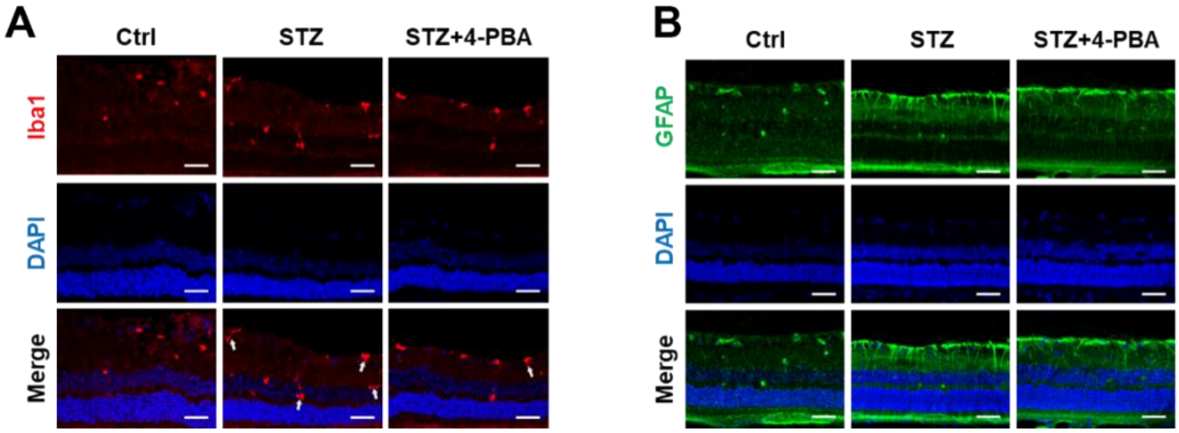

Figure S2. (A) Immunofluorescence staining for Iba1 (red) and DAPI (blue) was performed in rat retinal sections. White arrows indicate activated microglial cells with a rounded amoeboid morphology. **(B)** Immunofluorescence staining for GFAP (green) and DAPI (blue) was performed in rat retinal sections. Scale bars = 50 μm.
